# Exosomes Interactions with the Blood–Brain Barrier: Implications for Cerebral Disorders and Therapeutics

**DOI:** 10.3390/ijms242115635

**Published:** 2023-10-26

**Authors:** Zaynab Osaid, Mohamed Haider, Rifat Hamoudi, Rania Harati

**Affiliations:** 1 Department of Pharmacy Practice and Pharmacotherapeutics, College of Pharmacy, University of Sharjah, Sharjah P.O. Box 27272, United Arab Emirates; u20105690@sharjah.ac.ae; 2 Research Institute for Medical and Health Sciences, University of Sharjah, Sharjah P.O. Box 27272, United Arab Emirates; mhaider@sharjah.ac.ae; 3 Department of Pharmaceutics and Pharmaceutical Technology, College of Pharmacy, University of Sharjah, Sharjah P.O. Box 27272, United Arab Emirates; 4 Clinical Sciences Department, College of Medicine, University of Sharjah, Sharjah P.O. Box 27272, United Arab Emirates; rhamoudi@sharjah.ac.ae; 5 Division of Surgery and Interventional Science, University College London, London W1W 7EJ, UK

**Keywords:** Blood–Brain Barrier (BBB), neurovascular unit (NVU), exosomes, extra cellular vesicles (EV), brain tumors, neuroinflammation, neurodegenerative diseases

## Abstract

The Blood–Brain Barrier (BBB) is a selective structural and functional barrier between the circulatory system and the cerebral environment, playing an essential role in maintaining cerebral homeostasis by limiting the passage of harmful molecules. Exosomes, nanovesicles secreted by virtually all cell types into body fluids, have emerged as a major mediator of intercellular communication. Notably, these vesicles can cross the BBB and regulate its physiological functions. However, the precise molecular mechanisms by which exosomes regulate the BBB remain unclear. Recent research studies focused on the effect of exosomes on the BBB, particularly in the context of their involvement in the onset and progression of various cerebral disorders, including solid and metastatic brain tumors, stroke, neurodegenerative, and neuroinflammatory diseases. This review focuses on discussing and summarizing the current knowledge about the role of exosomes in the physiological and pathological modulation of the BBB. A better understanding of this regulation will improve our understanding of the pathogenesis of cerebral diseases and will enable the design of effective treatment strategies.

## 1. Introduction

The Blood–Brain Barrier is a semi-permeable structural and functional interface located at the brain microvessels that separates the circulatory system and the brain [1]. The barrier regulates the bidirectional exchange between the blood and the brain, where it controls the transport of ions, micro, and macromolecules. Its pivotal role also includes shielding the brain from endogenous and foreign toxins, thereby ensuring the homeostatic balance essential for proper brain activity [2]. Any disruption in its structural and functional integrity can subject the brain to a spectrum of pathological conditions [3]. Structurally, the BBB is a complex multi-cellular assembly composed of brain microvascular endothelial cells (BMECs), glial cells (astrocytes and microglia), pericytes, and a basement membrane [4,5]. Central to this assembly are the BMECs, which are tightly interconnected via three types of junctional complexes: tight junctions (TJs), adherent junctions (AJs), and gap junctions (GJs). TJs are the primary proteins restricting paracellular transport between BMECs, AJs (or cadherins) facilitate BMEC cohesion [6], and GJs are channel-like structures that enable BMEC intercellular communication. Any decreased expression of these junctional complexes renders the BBB more permeable [3]. Although the BMECs are key components forming the barrier, communication between the different cell types is required to reinforce the barrier’s integrity [7].

Exosomes were discovered in the late 1980s and were initially considered cellular debris [8]. They are now recognized as a subclass of vesicular bodies with diameters ranging from 40 to 150 nm. These cup-shaped particles are released into various body fluids by almost all cell types [9,10]. They fall under the broader umbrella of extracellular vesicles (EVs), a larger group of heterologous biological nanoparticles, including apoptotic bodies and microvesicles. Exosomes have gained attention for their role in intercellular communication, facilitated by their capacity to transport an array of bioactive molecules, thereby mediating a wide range of physiological processes [10]. Exosome content varies depending on the originating cells and may include nucleic acids, proteins, and lipids [9]. Once released, exosomes travel, intravasate, or extravasate to deliver their content to nearby or distant cells, affecting the functionality of the receiving cell. Exosomes are considered a promising drug delivery vehicle and therapeutic target because of their favorable physiochemical and biological attributes, including biocompatibility, stability, ability to penetrate biological barriers (including the BBB), minimal toxicity, and low immunogenicity [11]. Recently, the release of exosomes by different cell types in the CNS highlighted their ability to regulate various BBB-associated phenomena, such as tumor dynamics [12], angiogenesis processes [13], and immune responses [14].

Exosomes are essential for intercellular communication and affect both physiological and pathological aspects of the BBB. Their content and secretion have implications on the onset, progression, or suppression of several brain pathologies [15,16]. The purpose of this review is to discuss and summarize the existing literature on the role of exosomes in the physiological and pathological regulation of the BBB. This could provide insights into the pathogenesis of various conditions and pave the way for the development of improved therapeutic strategies.

## 2. Exosomes Biogenesis

### 2.1. Exosomes Biogenesis

**Figure 1:**
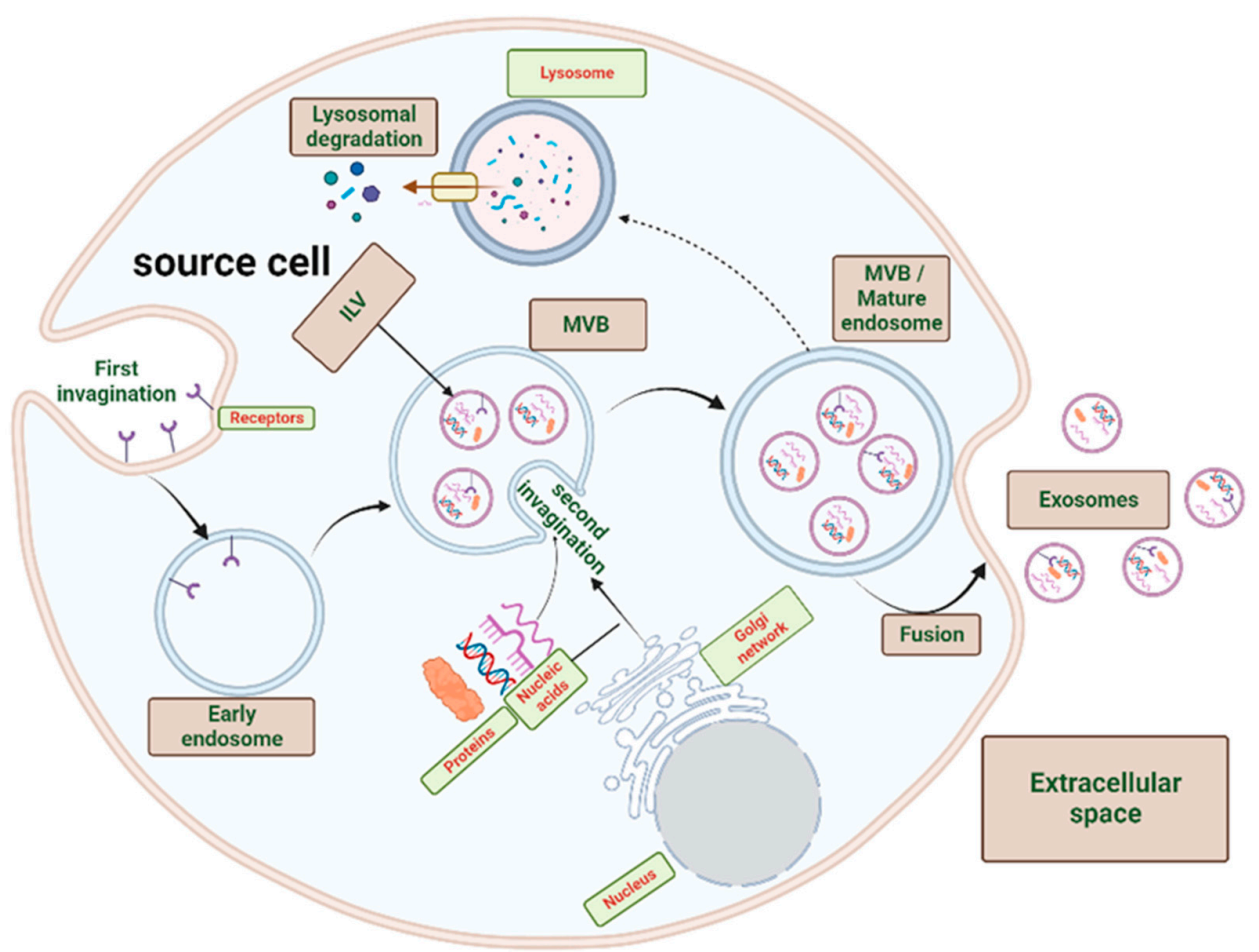
Exosome biogenesis. The early endosome is formed by an initial invagination of the cell plasma membrane into the cytosol. The intraluminal vesicles (ILV) are formed by a second invagination of this early endosome membrane. A multivesicular body (MVB) harbors the ILVs and is referred to as a mature endosome. Following cargo installation within the MVB ILVs, the MVB will be subjected to one of two fates: fusion with the plasma membrane and exocytosis of exosomes or lysosomal fusion and degradation. The figure was created with BioRender.

Exosomes are vesicles of endosomal origin. The early endosome is formed by an initial invagination of the cell plasma membrane into the cytosol [17]. The intraluminal vesicles (ILV) are formed by a second invagination of this early endosome membrane, and when these compartments are released into the extracellular space, they are designated as exosomes. A multivesicular body (MVB) harbors the ILVs and is referred to as a mature endosome [18,19]. Following cargo installation within the MVB ILVs, the MVB undergoes one of two potential fates: either fusion with the plasma membrane and exocytosis of exosomes or lysosomal fusion and degradation (Figure 1) [20].

The endosomal sorting complex required for transport (ESCRTs) coordinates MVB biogenesis and is important in ILV formation. ESCRTs are composed of four complexes (ESCRT-0 through III). ESCRT-0 is recruited to sort ubiquitinated proteins to the endosomal membrane; it then interacts with ESCRT-I and -II to aid in the budding of ubiquitinated protein vesicles; the resulting complex binds with ESCRT-III and forms the ILVs within the MVB [21,22]. The exosomal protein ALIX is an accessory protein in the ESCRT machinery involved in ILV budding and cargo sorting [23].

On the other hand, ESCRT-independent mechanisms have emerged as an alternative pathway in exosome formation and content segregation; the most studied mechanism is ceramide-dependent. Ceramide is a sphingolipid that forms raft structures, or microdomains, that can entrap various molecules. It can induce endosomal membrane inward curvature and ILV budding [24]. The presence of sphingomyelinase 2, an enzyme required for forming ceramide from sphingomyelin, is necessary for this process [25].

Despite advancements in our comprehension of exosome biogenesis, further investigation is still needed to better understand the exact mechanism that mediates exosome biogenesis and cargo sorting, which results in exosome heterogeneity.

### 2.2. Exosomal Content

**Figure 2:**
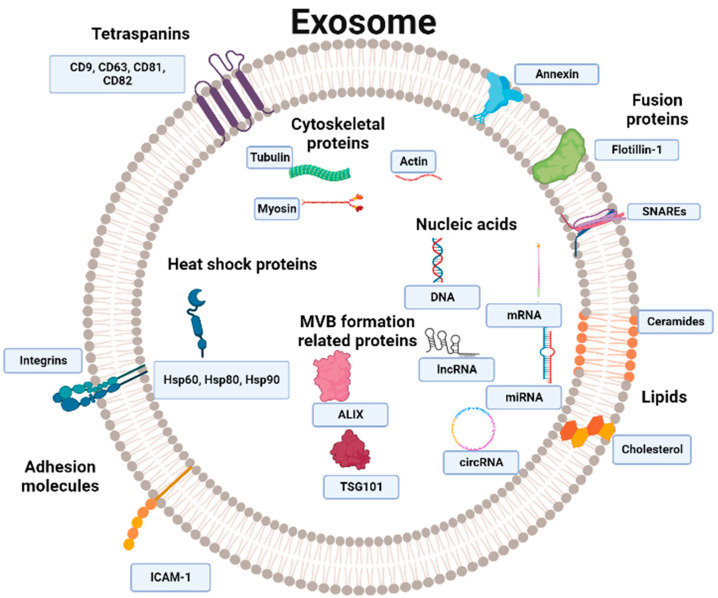
Exosomes content. Exosomes contain tetraspanins such as CD9, CD63, CD81, and CD82. Other exosomal proteins are flotillin and annexin Rab GTPases and SNARE fusion proteins, as well as heat shock proteins (Hsp) such as Hsp60, Hsp80, and Hsp90. Exosomes also harbor multivesicular bodies and MVB-formation-related proteins such as TSG101 and ALIX. Exosomes are also loaded with other types of proteins, some of which are involved in cytoskeleton formation, such as actin, tubulin, and myosin, and some adhesion molecules, such as ICAM-1 and integrins. Exosomes also carry some lipids, including cholesterol and ceramides. Exosomes conserve a diverse profile of nucleic acids, including DNA, messenger RNA (mRNA), microRNA (miRNA), long non-coding RNAs (lncRNA), and circular RNAs (circRNAs). The figure was created with BioRender.

Exosomes are enriched with different proteins, nucleic acids, and lipids, reflecting the molecular fingerprint of their parent cell (Figure 2). Among their protein constituents, tetraspanins, transmembrane proteins such as CD9, CD63, CD81, and CD82 are important exosomal surface proteins. These proteins are integral to the loading of exosome receptors and signaling molecules at the plasma membrane, facilitated by the formation of tetraspanin-enriched microdomains [26]. Exosomes also feature other proteins, such as flotillin and annexin fusion proteins [27], as well as heat shock proteins (Hsp) such as Hsp60, Hsp80, and Hsp90. HSPs ensure proper protein folding and adapt the exosome to the extracellular space [28]. Hsp90 was found to facilitate the fusion between the plasma membrane and MVB [29]. Exosomes also contain MVB-formation-related proteins such as TSG101 and ALIX. Most of the proteins mentioned above are used as markers in the characterization of exosomes [27].

Additionally, exosomes incorporate proteins involved in cytoskeleton formation, such as actin, tubulin, myosin, and some adhesion molecules [30]. The release of exosomes by exocytosis is mediated by the interactions of Rab GTPases and SNARE proteins with the MVB [31].

Furthermore, exosomes contain proteins specific to the originating cell type; for example, T-cell exosomes express CD3 protein [32]. Likewise, the antigen presentation protein, namely the major histocompatibility complex (MHC), is present in dendritic cell exosomes [33].

Even though exosomes are lipid-rich, the precise composition and role of these lipids require further investigation. Notably, exosomes contain cholesterol, ceramides, lyosbisphosphatidic acid (LBPA), bismonoacylglycerol phosphate (BMP), phosphatidylserine, and glycosphingolipids [34]. According to previous studies, ILV cholesterol content is higher in the early stages of MVB maturation, whereas BMP is more abundant in the later stages, aiding in exosome formation [35]. Lipids provide structural support for exosomes and impact their content and release; external conditions influence exosome lipid composition. For instance, a study demonstrated that treating PC3 cells with hexadecylglycerol increases the ether lipid level in the cells, subsequently augmenting the quantity of released exosomes and their ether lipid content [36].

Exosomes also contain a diverse profile of nucleic acids, including a range of different DNA moieties, messenger RNA (mRNA), and a plethora of non-coding RNAs (ncRNA), such as microRNA (miRNAs), long non-coding RNAs (lncRNAs), and circular RNAs (circRNAs). This complex exosomal cargo can modulate gene expression in recipient cells, affecting their biological processes by triggering downstream signalling pathways [37,38].

## 3. Transport of Exosomes through the Blood–Brain Barrier

The BBB serves as a selective filter, permitting the transit of specific molecules based on their physicochemical characteristic through passive diffusion or active transport. Alvares-Erviti et al. were among the first to highlight the ability of exosomes to cross the BBB and deliver their cargo to the brain [39]. Exosome transport through the BBB has been reviewed elsewhere [40].

Interestingly, exosomes demonstrated bidirectional movement across this barrier. A study confirmed the presence of proteins specifically released by astrocytes in the blood of rats within exosomes, confirming the ability of exosomes to travel from the CNS to the peripheral circulation [41]. Several hypothetical mechanisms by which exosomes could interact with the BMEC and cross the barrier have been suggested by researchers. These include fusion with the plasma membrane, paracytosis, transcytosis, and the engulfment of exosomes via micropinocytosis [42,43,44]. The different pathways exosomes used to move across the BBB are summarized in Table 1. Among these, transcytosis emerges as the predominant pathway of exosome transport across the BBB. Research has shown that synuclein-containing exosomes released by erythrocytes can cross the BBB via adsorptive-mediated transcytosis [44]. Post-transcytosis, the exosomes are either subjected to degradation or inserted within endosomes after the formation of an MVB [45]. A study on the endocytosis-mediated transcytosis route of exosomes demonstrated that subsequent to internalization by BMEC, exosomes are localized within endosomes and then released across the BBB. The study further illustrated that endocytosis of exosomes at the BBB is clathrin- and caveolae-dependent [46]. A study by Banks et al. showed that exosomes may be partially sequestered within BMEC, affecting endothelial function [47].

Despite these insights, more research is still required to uncover the diverse mechanisms exosomes use to traverse the BBB, considering their cell of origin and different environmental conditions.

## 4. Physiological Regulation of the Blood–Brain Barrier by Exosomes

Various mechanisms rigorously regulate the cellular release of exosomes within the CNS [49]. Specifically, exosome release from neurons is controlled by glutamatergic synaptic activity, facilitated by calcium influx via N-methyl-D-aspartate (NMDA) and amino-3-hydroxy-5-methyl-4-isoxazolepropionic acid (AMPA) receptors [50]. Additionally, 5-HT plays a role in exosome release from primary microglial cells by interacting with serotonin (5-HT), 5-HT4, and 5HT2a,b receptors, thereby increasing cytosolic calcium release [51]. In disease conditions, it has been shown that inflammatory stimuli induce exosome release from astrocytes, endothelial cells (EC), and microglia [52,53,54,55,56].

Few studies investigated the exosome-mediated regulation of the BBB. The content of exosomes derived from BMEC under normal physiological conditions was investigated recently. Using proteomic analysis, exosomes were found to be enriched with histones, ribosomal, and adhesion proteins [57].

The discovery of nucleic acids and proteins within exosomes highlighted their importance as mediators for cell-to-cell communication in both normal and pathological conditions. The exosome-facilitated communication has been demonstrated to influence the BBB phenotype. Supporting this notion, a zebrafish model was used to test the involvement of neuronal exosomes in regulating the BBB. Results showed that neurons release exosomes containing miR-13 upon internalization by BMEC, leading to the upregulation of TJ (VE-cadherin) expression, thereby reinforcing BBB integrity [58]. Similarly, astrocytes’ cross-talk with the BMEC through the release of paracrine factors regulates the properties of the BBB [59]. An investigation on the effect of exosomes derived from mice astrocytes on the permeability of the barrier showed that exosomes from healthy astrocytes preserved BBB integrity by upregulating the expression of TJs and enhancing the electrical resistance of BMEC [60].

Establishing a firm BBB requires the precise alignment of the BMEC, ensuring their TJs interconnect to occlude the paracellular space. Therefore, the intercellular communication between the BMEC was studied to understand the morphogenesis of the BBB [61]. Virgintino et al. reported the role of EVs in modulating the BMEC-emitted filopodia processes during human fetal brain vasculature development, postulating their contribution to vascular morphogenesis via cell-to-cell communication [62]. Supporting this, recent papers by Fisher et al. have investigated the molecular basis underpinning the development of the BBB by focusing on the underlying nanoscale network involved. Their results identified the presence of nanovesicles, including exosomes, on the plasma membrane of BMEC, suggesting their involvement in paracrine signaling. This signaling is believed to generate a chemoattractant gradient, inducing nanotube formation by fusing neighboring exosomes. These nanotubes fill the paracellular gaps between BMEC, connecting them and facilitating the alignment of their TJs, therefore enhancing the integrity of the barrier [61,63].

Exosome research is witnessing swift advancements, paving the way for a more profound understanding of exosomes and their function in the physiological regulation of the BBB. This enhanced understanding will further elucidate the intricacies of intercellular communication pathways. Gaining insights from these pathways holds the potential for developing novel therapeutic approaches to address cerebral disorders.

## 5. Regulation of the Blood–Brain Barrier by Exosomes in Brain Diseases

### 5.1. Brain Glioma

Brain tumor progression causes a dynamic change in the morphologic properties of the BBB, resulting in a disrupted barrier known as the blood–tumor barrier [64,65]. A significant characteristic of the altered barrier is its high permeability compared to a healthy BBB [66]. Communication between matrix cells and cancer cells in the tumor microenvironment (TME) using non-cell material is vital for tumor evolution [67]. Several studies suggest that tumor-derived exosomes play an essential role in reshaping the brain microenvironment. These exosomes favor tumor growth, angiogenesis, and metastasis by serving as transporters of tumor cell bioactive cargo that facilitates these processes [13,68,69].

Glioma defines a set of primary brain tumors that emerge from glial or supporting cells. These tumors are categorized according to the cell of origin into oligodendrogliomas, astrocytomas (glioblastomas), ependymomas, and mixed gliomas [70,71]. Gliomas are very heterogenous and vary in their invasiveness [72]. Glioblastoma (GBM) stands out as a particularly aggressive and rapidly proliferating tumor often associated with a poor prognosis. The estimated survival period post-diagnosis is slightly over one year [73,74,75,76]. GBM is notably one of the most vascularized tumors. Its progression depends on angiogenesis, which is supported by the overexpression of vascular endothelial growth factor (VEGF) in these tumors [77]. Oxygen deprivation in the tumor microenvironment, known as hypoxia, is a significant driver of angiogenesis. The accelerated growth of GBM eventually leads to tumor regions with extreme hypoxia stemming from increased oxygen demand [78]. It has been established that hypoxic conditions compromise the integrity of the BBB [79,80]. Specifically, in GBM, the disruption occurs at the level of TJs, resulting in a leaky BBB [80]. However, the pathogenesis of the BBB breakdown in GBM requires further investigation.

Multiple studies have highlighted that GBM cells release exosomes laden with a variety of pro-angiogenic factors, including VEGF [81,82]. According to one study, GBM cells release exosomes that can induce the lumen formation, growth, and migration of BMEC. This is attributed to the combined effects of pro-angiogenic factors, proteolytic enzymes, and the angiogenesis-associated chemokine receptor CXCR4 [83].

Zhao et al. investigated the impact of exosomes released by the GBM on BBB permeability under hypoxic conditions. It was demonstrated that hypoxic GBM-derived exosomes increase the permeability of human brain microvascular endothelial cells (HBMEC) in vitro. They reported that barrier disruption was mainly derived from the VEGF-A content of the exosomes. Further analysis confirmed the detrimental effects of these exosomes on HBMEC TJs, as evidenced by the decreased expression of occludin and claudin-5. Similar results were observed in in vivo experiments [82].

**Figure 3:**
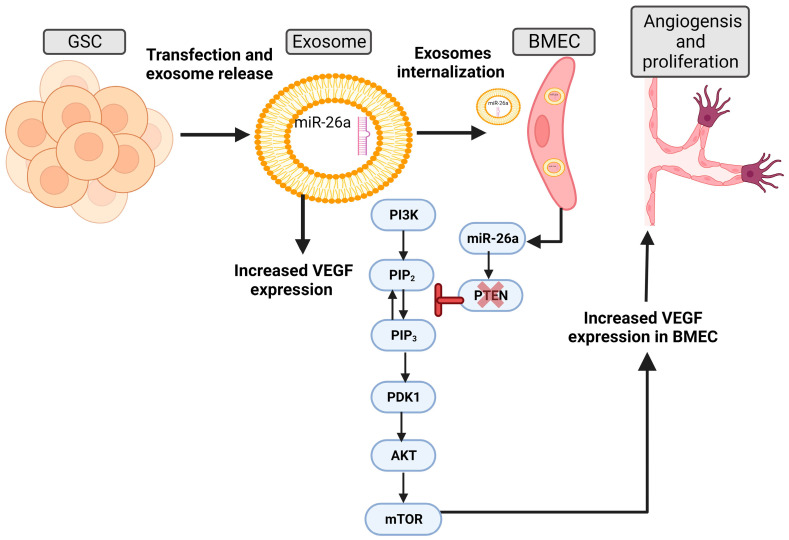
Effect of exosomes derived from glioma stem cells and containing miR-26a on the BBB. Increased vascular endothelial growth factor (VEGF) expression in exosomes derived from glioma stem cells (GSC) pre-transfected with miR-26a led to further VEGF upregulation in human brain microvascular endothelial cell (HBMEC) after uptake. miR-26a targets the tumor suppressor PTEN and reduces its expression; the result of this regulation is the activation of the PI3K/Akt signaling pathway to stimulate angiogenesis and proliferation of HBMEC. The figure was created with BioRender.

Glioma stem cells (GSCs) enhance the aggressiveness of GBM and promote angiogenesis [84,85]. Several studies probed the role of GSC-derived exosomes in the development of angiogenesis in HBMEC, particularly focusing on the effect of their miRNA cargo [13,68]. Improved VEGF expression in GSC exosomes transfected with miR-21 or miR-26a led to further VEGF upregulation in HBMEC after uptake. This also increased HBMEC’s angiogenic capacity, which had been significantly reduced following VEGF knockdown. The data suggest that the angiogenic capacity of HBMECs was augmented via the miR-21/VEGF signal, with the downstream effect being attributed to the activation of VEGF receptor type 2 in HBMECs [13]. While miR-26a targets the tumor suppressor PTEN and reduces its expression, the outcome of this regulation is the activation of the PI3K/Akt signaling pathway to stimulate the angiogenesis and proliferation of HBMEC [68] (Figure 3).

Exosomes released by glioma cells containing long intergenic non-coding RNAs (lincRNAs) internalized by HBMECs have also been linked to the induction of angiogenesis [86]. Research showed that the exosomal transfer of linc-POU3F3 to HBMECs stimulates their proliferation and tube formation capability. This enhancement is attributed to the upregulation of angiogenesis-associated genes and proteins [86].

Exosomes’ ability to penetrate the BBB, biocompatibility, and low immunogenicity have positioned them as promising vehicles for drug delivery [11]. In a recent study, exosomes derived from BMECs were used to inhibit the proliferation of gliomas by overexpressing a tumor suppressor gene, esophageal cancer-related gene-4 (ECRG4). After transfection of BMECs with a lentivirus expressing ECRG4 gene, the resultant exosomes (ECRG4-EX) were internalized by glioma cells. This uptake increased the intracellular ECRG4 levels and resulted in a notable inhibition of cell viability, proliferation, angiogenesis, and inflammation. The underlying mechanism of these observed effects was associated with the ability of ECRG4-EX to suppress the level of angiogenesis and inflammation-related factors in glioma cells. In vivo, similar results were obtained and proved to be attributed to p38-MAPK signaling pathway inhibition [87].

Recently, it was shown that glioma-derived exosomes can increase BBB fluidity by increasing lipocalin-2 (LCN2) gene expression, which codes for a transporter protein in HBMEC. This upregulation was mediated through the activation of the JAK-STAT3 pathway. This mechanism was suggested to be used by GBM-derived exosomes to manipulate the BBB [88].

Numerous researchers have investigated the potential of exosomes as nano-delivery carriers for anti-cancer medications in glioma therapy. For example, drugs such as doxorubicin (DOX), typically unable to traverse the BBB, were effectively encapsulated in exosomes using microfluidic techniques to target gliomas specifically [89]. A different research study indicated that exosome-coated nanoparticles containing the drug DOX can cross the BBB following internalization by BMECs. Consequently, there was an observed augmentation in apoptosis and a concomitant decrease in the proliferation of GBM cells [90]. Lee et al. employed the intrinsic tropism of GBM exosomes loaded with selumetinib. The authors propose that GBM exosomes containing sulmetinib target parent cells while minimizing harm to healthy cells, suggesting potential possibilities for GBM treatment [91].

Moreover, the surface functionalization of exosomes has been explored. It was observed that EVs incorporating methotrexate (MTX) modified with therapeutic peptides [Lys-Leu-Ala (KLA)] and targeted low-density lipoprotein (LDL) peptides achieved increased uptake by the GBM cell line U87 and improved penetration across the BBB as well as within three-dimensional GBM spheroids enhancing the therapeutic effectiveness of MTX by this modification [92]. In the context of exosomes specifically, research has demonstrated that exosomes derived from embryonic stem cells and engineered with the Cyclo (Arginine-Glycine-Aspartic-D-Tyrosine-Lysine) peptide displayed enhanced specificity for glioma targeting and demonstrated increased efficacy in improving the antitumor effect of Paclitaxel [93].

Different investigators utilized exosomes to tackle temozolomide (TMZ) resistance in glioma treatment [94,95]. For instance, Wang et al.’s research used reassembled exosomes from homologous glioma cells to incorporate dihydrotanshinone and TMZ. In the same way, John added heme oxygenase-1-specific short peptides (HSSP) to exosomes from bone marrow mesenchymal stem cells and then loaded them with TMZ and siRNA. The results of these two studies showcased their efficacy in overcoming BBB and their effective accumulation in glioma lesions, diminishing treatment resistance, thus suggesting a possible anti-cancer effect [94,95].

### 5.2. Metastatic Brain Tumors

Metastatic brain tumors are the most prevalent intracranial malignant neoplasms that develop secondary to a range of primary tumors [96]. The most common types of cancer that metastasize to the brain are lung cancer, breast cancer, and melanoma, in descending order of frequency [97,98]. Brain metastasis (BM), an end-stage of cancer, is associated with a poor prognosis [99,100]. The development of BM depends on intracellular communication through secreted factors, mainly exosomes, between metastatic tumor cells and the brain microenvironment [101,102]. The transgression of the BBB by cancer cells and the subsequent establishment of neoplasms at secondary organ sites are triggered when tumor cells recognize and adhere to components of the vascular membrane [103]. This interaction alters BBB integrity and contributes to BM [104]. Exosomes are involved in the regulation of multiple features of malignancy in tumors [13,69]. Nevertheless, a clear understanding of their role in BM is still lacking. In this perspective, the role of exosomes in the transmigration of cancer cells through the BBB has been the focus of several studies [12,105]. In addition, several studies showed the metastatic role of micro-RNAs in the extravasation of cancer cells through the BBB [106,107,108,109,110,111,112]. However, the role of exosomal micro-RNAs in this context merits further study.

#### 5.2.1. Metastatic Breast Cancer

Breast cancer is a prevailing form of cancer and is a primary contributor to cancer-related death in females [113]. Metastasis is a major factor contributing to morbidity and mortality in breast cancer patients [114]. The involvement of exosomes in the transmigration of breast cancer cells across the BBB and the establishment of BM is an area of active research.

Evidence suggests that the miRNA content of exosomes contributes to the alteration of the BBB integrity. In this context, a study examined the role of exosomes containing miR-105 secreted from the MDA-MB-231 breast cancer cell line as a migratory regulator in HBMEC. Results showed that exosomal miR-105 reduced the expression of the TJ protein ZO1 in HBMEC upon uptake. This led to increased BBB permeability, promoting metastasis both in vitro and in vivo [69]. Similarly, Tominaga et al. identified a novel mechanism by which EVs, including exosomes, mediates BM and results in the breakdown of the BBB. The study demonstrated that the uptake by BMEC of exosomes derived from brain metastatic breast cancer cells and containing miR-181c changes the localization of several TJs and actin filaments by inducing the downregulation of the PDPK1 gene in BMEC. The outcome is elevated BBB permeability, thereby promoting metastasis [12].

Similar to exosomal miRNAs, the impact of exosomes carrying lncRNAs on breast cancer BM was investigated. The transfer of lncRNA GS1-600G8.5 from triple-negative breast cancer exosomes into BMECs increased the BBB permeability, whereas the silencing of GS1-600G8.5 preserved the barrier integrity. The modulatory effect of exosomal GS1-600G8.5 on the BBB was denoted by targeting its TJs [115].

A limited number of studies have probed the role of exosomes secreted by breast cancer cells in establishing a pre-metastatic niche, thereby influencing the BMECs of the BBB to promote metastasis [116,117]. Exosomes derived from brain metastatic breast cancer cells contain cell migration-inducing hyaluronan binding protein (CEMIP), which increases vascular co-option and elevates the brain metastatic potential of tumor cells in vitro. The loss of exosomal CEMIP diminishes tumor cells and vasculature interaction, thus impeding their invasive capacity in the brain. In vivo analyses revealed that exosomal CEMIP alters the morphogenesis of BMEC due to its ability to elicit a proinflammatory phenotype in brain vasculature and microglial cells, thereby fostering tumor cell metastasis and cerebral colonization [117]. Interestingly, the overexpression of the enzyme tubulin tyrosine ligase such as 4 (TTLL4) in breast cancer cells increases the secretion of exosomes and accelerates the movement of secretory vesicles and MVBs from TTLL4+ cells. It was also shown that exosomes produced by TTLL4 overexpressing cells promote the adhesion of breast tumor cells to HBMEC and enhance BBB permeability by transporting proteins involved in metastasis and invasion [116].

In controversial research, Fazakas et al. used atomic force microscopy (AFM) to assess the de-adhesion strength of breast cancer cells to BMEC following incubation with exosomes derived from breast adenocarcinoma. The study concluded that pre-treated BMEC with exosomes from breast adenocarcinoma exhibited lower de-adhesion strength than untreated BMEC. This suggests that the formation of adhesion to the BMEC of the BBB might be independent of the presence of breast tumor-derived exosomes [118]. Further investigations are imperative to better understand the role of exosomes in brain metastasis.

#### 5.2.2. Metastatic Lung Cancer

Lung cancer ranks among the most prevalent types of cancer, accounting for a high fatality rate worldwide [119]. Between 10% and 25% of lung cancer patients present with BM at diagnosis, and this incidence can rise to 50% as the disease progresses [120]. However, the mechanisms associated with the changes in BBB permeability during lung cancer BM are still unclear. Investigating the involvement of exosomes in lung cancer BM development through BBB disruption provides new insights into the treatment of lung cancer. Gan et al. showed that lung cancer cell exosomes induce the release of DKK1 from HBMEC and promote a pro-tumorigenic phenotype of microglia conducive to brain invasion [121]. In accordance with this, it was also elucidated that exosomes derived from a highly metastatic lung cancer cell line and enriched with miR-550a-3-5p significantly repressed the migration and cell viability of HBMECs while promoting apoptosis. Yes-associated protein 1 was reported as the gene target of miR-550a-3-5p responsible for these effects [16].

In addition, it was shown that exosomal lncRNA (LINC01356 and Lnc-MMP2-2) from highly metastatic non-small cell lung cancer (NSCLC) can remodel the BBB. They achieve this by downregulating the expression of the BMEC TJs, thereby compromising the integrity of the BBB and facilitating BM [122,123]. TGF-β1 mediates the release of NSCLC exosomes carrying Lnc-MMP2-2, which in turn acts as a miRNA sponge for miR-1207-5p in BMECs, upregulating erythrocyte membrane protein band 4.1 Like 5 (EPB41L5). This induces endothelial-to-mesenchymal transition and concurrently suppresses BMEC TJs, creating a favorable microenvironment for BM [123].

Small cell lung cancer (SCLC) accounts for 30% of BM cases and is recognized as the most aggressive lung cancer subtype [124,125]. Xu et al. showed that exosomes from HBMECs elevate the level of S100A16 in SCLC, creating a protective effect that enhances BM and apoptosis resistance in SCLC. Further investigations revealed that exosomal S100A16 participates in preserving the mitochondrial membrane potential, thus ensuring the survival of SCLC cells in the cerebral milieu. These results indicate the potential of exosomes derived from BMECs in BM genesis [126].

#### 5.2.3. Other Types of Metastatic Cancer

Exosomes impact on BBB breaching extends to leukemia and melanoma. BMEC pre-treated with exosomes from precursor B cell acute lymphoblastic leukemia (BCP-ALL) contributed to the infiltration of leukemia cells through the BBB. Additionally, cultured astrocyte internalization of BCP-ALL exosomes increased VEGF-AA production and abrogated BBB integrity [105]. In melanoma, CD46 was reported as a major receptor facilitating the internalization of melanoma exosomes by HBMEC [42]. By using a BBB chip, malignant melanoma-derived exosomes increased BBB permeability by downregulating the expression of ZO1 and VE-cadherin and promoting glial activation [127]. The effect of different exosomal contents derived from different metastatic cancers on the BBB is summarized in Table 2.

Collectively, these studies underscore the involvement of exosomes in promoting the transmigration of cancer cells through the BBB by affecting its structural integrity. Therefore, exosomal content could be explored as a potential therapeutic target for treating different types of cancer.

### 5.3. Traumatic Brain Injury

Traumatic brain injury (TBI) is a significant global health concern, contributing to considerable mortality and morbidity rates [128,129]. TBI induces pathophysiological disruption in BBB permeability [130]. Recent evidence suggests that exosomes can alter the structure and functionality of the BBB post-TBI, providing an effective treatment method [131].

In recent years, human mesenchymal stem cell (MSC)-derived exosomes have emerged as a potential treatment for TBI [132]. Several studies have shown the efficacy of MSC exosomes in improving BBB stability post-TBI. In a set of investigations using the TBI swine model, Williams et al. demonstrated that early treatment with a single dose of MSC exosomes significantly reduced cerebral swelling and lesion size, with a subsequent decrease in intracerebral pressure and albumin extravasation [133,134,135]. This was associated with an upregulation of laminin, claudin-5, and ZO-1, signifying improved BBB integrity [133]. In a comparable design model of transcriptome alterations, a single dosage of MSC exosomes was shown to upregulate genes linked to BBB integrity and downregulate genes related to BBB dysfunction [134]. Additionally, in a seven-day survival model, early single-dose exosome treatment not only reduced the size of brain lesions but also suppressed inflammation and apoptosis, enhanced neural plasticity, and lowered ICP levels, which are all indicative of improved BBB integrity [135].

Another type of progenitor cell, endothelial colony-forming cells (ECFCs), secretes EVs known to support angiogenesis and preserve BBB connectivity post-TBI [136,137]. In vitro studies have shown that ECFC-derived exosomes augmented EC migration and restored TJ protein expression, primarily via targeting the PTEN/AKT signaling pathway. In murine models of TBI, these exosomes exhibited robust BBB protective effects, characterized by decreased MMP-9 expression and the prevention of TJ protein degradation [138].

Exosomal miR-21 released by macrophages has been implicated in BBB deterioration. Ginsenoside Rg1, an active biomolecule derived from ginseng, demonstrated a protective effect by hindering the release of exosomal miR-21 to the brain. This intervention resulted in an accelerated proteolysis of MMPs and upregulated expression of TJs, thereby preserving BBB integrity. Such findings position ginsenoside Rg1 as a potential therapeutic candidate for TBI management [139].

### 5.4. Stroke

The destabilization of the BBB by triggering BMEC inflammation, dysfunction, and apoptosis are key pathophysiological features of a stroke [140,141]. Several studies have underscored the importance of preserving BBB function, highlighting its positive implications for post-stroke recovery [142,143]. Recently, interest has increased in the role of EVs in maintaining the barrier. Zhang et al. demonstrated that neural-progenitor-cell-derived EVs can reduce post-stroke BBB permeation by inhibiting the NF-κB signaling pathway in BMEC, thereby regulating the expression of ATP-Binding Cassette Transporter B1 and MMP-9 [144].

There is growing evidence that exosomes specifically affect BBB integrity in various ways post-stroke [15,145,146,147]. For example, exosomes had a protective effect on the BBB in a rat stroke model. Huang et al. elucidated that healthy exosomes obtained from healthy rat serum inhibited BMEC apoptosis and autophagy-related BBB breakdown while preserving claudin-5 and ZO-1 expression in experimental stroke both in vitro and in vivo [145]. Similarly, using the hypoxia/reoxygenation-injured BMECs model exosomes enriched with miR-132-3p from MSC lowered ROS production and BMEC apoptosis. These exosomes also sustained the TJs protein expression, offering dual benefits of BBB protection and antioxidant activity in vivo [146]. Moreover, exosomes treated with scutellarin were documented to protect BMECs by diminishing homocysteine-induced injury to TJ proteins and ROS-mediated oxidative stress on the BBB [147]. Collectively, these results demonstrated that exosome therapy improves BBB integrity in stroke.

In addition, BBB breakdown caused by stroke has been linked to astrocyte-released exosomes containing the lncRNA H19. These exosomes, bearing lncRNA H19, induce BBB dysfunction by depriving BMEC of oxygen and glucose as well as downregulating TJ protein expression through the lncRNA H19/miR-18a/VEGF signaling axis [15].

### 5.5. Neuroinflammation

Neuroinflammation occurs when an inflammatory threat triggers the brain’s innate immune system. As a result, leukocytes infiltrate the BBB, brain resident astrocytes and microglia are activated, and the release of pro-inflammatory mediators is stimulated. These events compromise the integrity of the BBB and further exacerbate neurological deterioration [148]. Some neuroinflammatory processes, both infectious and neurodegenerative in nature, appear to be modulated by exosomes [149]. The role of exosomes in BBB regulation in the context of neuroinflammation has been extensively studied. The data suggest that exosomes are released into the CSF by the choroid plexus epithelium in response to systemic or lipopolysaccharide (LPS)-induced inflammation. These exosomes Harbor proinflammatory miRNAs that traverse the BBB and induce a neuro-inflammatory response in brain astrocytes and microglia [7]. Additionally, exposure to tumor necrosis factor (TNF) has been shown to alter the proteomic content of BMEC-derived exosomes, incorporating proteins associated with inflammatory pathways [57].

LPS-induced neutrophils were shown to secrete exosomes containing miR-122-5p. This miRNA induces oxidative stress, apoptosis, and elevated permeability of BMECs by downregulating occludin expression [150]. While monocyte trafficking across the BBB is a standard physiological event, recent in vitro investigations highlighted the importance of exosomes in facilitating that process during inflammation. Exosomes from activated monocytes, carrying proinflammatory miRNAs, activate the NF-kB signaling pathway in BMECs. This activation results in altered inflammatory miRNA expression, which in turn promotes monocyte chemotaxis through the BBB by upregulating cytokines and adhesion molecules ICAM-1 and VCAM-1. Exosome secretion inhibition mitigated these effects [14]. Furthermore, the adhesion of leukocytes to the BBB is crucial in the developing of neuroinflammatory disorders [151]. BMEC-derived exosomes induce ectopic expression of claudin-5 on circulating leukocytes, acting as a temporary TJ bridge to BMEC. This interaction facilitates leukocyte trans-endothelial migration during neuroinflammatory episodes [152].

A growing body of literature demonstrates the role of pathogens in regulating the BBB through exosome secretion, thereby facilitating their neuroinvasive capabilities [153]. For instance, mast cell-derived exosomes have been implicated in exacerbating cerebral malaria in murine models. These exosomes compromise BBB integrity by downregulating TJs expression and activating brain vascular endothelial cells. This activation is evidenced by the increased expression of adhesion molecules ICAM-1 and VCAM-1, in turn amplifying the pro-inflammatory response in the affected mice [153].

It is important to note that HIV-1-associated neurocognitive disorders affect 50–60% of HIV-1 patients. This significant prevalence is attributed, in part, to BBB disruption and persistent neuroinflammation [154]. Exosomes isolated from HIV-infected cells contribute to BBB impairment by containing viral proteins such as Tat and Nef, which allow viral infiltration into the brain [155,156]. Latent HIV-1-infected immune cells release exosomes packaging Tat protein that increases mitochondrial hyperfusion by mediating the loss of phosphorylated dynamin-related protein 1 (p-DRP1) and downregulating phosphorylated endothelial nitric oxide synthase (p-eNOS) expression in BMECs, thus potentiating BBB damage [155]. Furthermore, exosomes secreted by Nef-transfected microglia and containing Nef protein induce inflammatory cytokine release from microglia. This inflammatory response, coupled with the downregulation of ZO-1, further deteriorates the BBB. Notably, blocking the production of these exosomes counteracted the associated BBB disruption [156].

Additionally, the BBB regulates the efflux of amyloid beta (Aβ) from the brain. Andras et al. showed that HIV-1 infection increases the release of Aβ-containing exosomes from BMEC. These exosomes are subsequently transferred to surrounding astrocytes and pericytes of the BBB, further amplifying its disruption [157,158]. Given these findings, further research is required to better understand the role of exosomes derived from pathogens in regulating the BBB.

### 5.6. Neurodegenerative Diseases

Neurodegenerative diseases refer to a group of debilitating neuronal conditions characterized by the progressive degeneration of the neurons of the CNS [159]. Alzheimer’s disease (AD) is the most prevalent among these disorders, primarily manifested as late-onset dementia [160]. Closely following AD in prevalence is Parkinson’s disease (PD), a neurodegenerative movement disorder primarily affecting motor neurons [161]. The progressive nature of these diseases is intrinsically linked to the deposition of cytotoxic protein aggregates that vary in composition depending on the disease. Specifically, AD is characterized by the accumulation of Aβ and tau proteins, while PD is marked by the accumulation of synuclein [159,162,163]. Due to their important role in intercellular communication, exosomes are suggested to be key effectors in the transport of these aggregates, potentially contributing to the development of these neurodegenerative conditions [163].

The BBB efflux function is critical for the normal clearance of Aβ from the brain, a process integral to maintaining neural hemostasis [164]. Dysfunctional BBB dynamics have been implicated in the pathogenesis of AD [165]. Empirical evidence suggests that BBB impairment causes Aβ accumulation by enhancing its production and altering its regular transport through the BBB [166,167]. Multiple studies examined the role of exosomes in BBB disruption. Notably, in vitro studies have demonstrated the potential of human neuronal stem cell (NSC)-derived exosomes to ameliorate AD-induced BBB leakage [168].

In addition, it is known that P-glycoprotein (P-gp), an ABC transporter situated on the surface of BBB BMECs, aids in the efflux of Aβ [169]. Intriguingly, a study presented evidence that HBMEC-derived exosomes, endowed with P-gp, significantly improved cerebral clearance of Aβ. This was achieved by effectively increasing the expression of P-gp on the HBMEC of the BBB, thereby enhancing the transport of Aβ out of the brain and potentially mitigating cognitive dysfunction in AD mice [149]. Another study investigated the effect of using curcumin-loaded exosomes derived from curcumin-treated mouse macrophage cells on improving cognitive function in vitro and in vivo. Due to the inherent affinity of these exosomes for lymphocyte-function-associated antigen and ICAM-1, they efficiently traversed the BBB, leading to increased cerebral curcumin accumulation in murine models [170].

In the context of PD, recent findings suggest that exosomes can be a principal element in mediating the abnormal cerebral accumulation of synuclein. Matsumoto et al. used peripherally administered LPS to induce systemic inflammation in murine models, and they observed a provoked increase in BBB permeability. This was characterized by enhanced adsorptive-mediated transcytosis of EVs, including exosomes loaded with synuclein produced by erythrocytes, potentially exacerbating PD pathogenesis [44].

Moreover, exosomes derived from human umbilical cord MSCs have been shown to overcome the limited BBB permeability to stem cells and prevent dopaminergic neuron loss in a PD rat model [171]. Another innovative strategy harnessed the transferrin–transferrin receptor interaction between unmodified mouse blood exosomes that express transferrin and BBB HBMECs to facilitate the BBB transport of dopamine-loaded exosomes. Using native transferrin-expressing mouse blood exosomes, dopamine was efficiently transported across the BBB via transferrin receptor-mediated endocytosis [172]. Although no clinical applications for exosomes have been established yet, few clinical trials are investigating the usage of exosomes as CNS disease therapeutics. These clinical trials are summarized in Table 3.

Collectively, these findings reveal the multifaceted roles of exosomes in the course of neurodegenerative diseases. However, the exact function of exosomes in neurodegenerative diseases or whether they are implicated in the degeneration process warrants further investigation.

## 6. Conclusions and Prospects

Exosomes exert diverse regulatory effects on the BBB, contingent upon their molecular constituents and the originating cell type. These EVs could promote or suppress disease pathogenesis. Indeed, exosomes can enhance tumor growth and disrupt the integrity of the BBB through mechanisms such as facilitating angiogenesis or downregulating the TJs of BMECs. Similarly, exosomes can increase the permeability of the BBB in cases of neuroinflammation. On the other hand, exosomes have therapeutic potential due to their capacity to facilitate BBB regeneration after TBI and stroke. They also show promise in ameliorating disruptions of the BBB, thus addressing cognitive impairments associated with AD and PD. Significant interest in exosomes as disease biomarkers and potential therapeutic targets has emerged in recent years. However, to harness their full therapeutic potential, it is imperative to determine the precise mechanisms governing their BBB transport. Furthermore, gaining insights into the intricacies of exosomal cargo sorting will be essential for tailoring their therapeutic payload and optimizing their clinical efficacy. Exosome isolation techniques, dosage determination, potential side effects, and administration routes are some of the many challenges that remain to be addressed. In conclusion, while exosomes offer a promising strategy to treat BBB-associated disorders, rigorous research endeavors are essential to translate these preliminary findings into clinical exosome-centric therapeutic strategies.

## Figures and Tables

**Table 1:** The different pathways used by exosomes to move across the Blood–Brain Barrier.

Transport Pathway	Mode of Transport	Energy Requirement	Reference
Fusion with the plasma membrane	Movement is achieved through physical interaction between the exosome and the cell plasma membrane	Low	[48]
Paracytosis	Movement of exosomes between the BMEC	Low	[43]
Transcytosis	Movement of exosomes across the BMEC	High	[45,46]
Micropinocytosis	The inward curvature of the cellular membrane and engulfment of exosomes	High	[43]

**Table 2:** The effect of different exosomal content derived from different metastatic cancers on the Blood–Brain Barrier.

Type of Metastatic Cancer	Exosomal Content	Effect on the BBB	In-Vivo/In-Vitro	Reference
Breast cancer	miR-105	Reduce the expression of TJs at BMEC	in-vitro and in-vivo	[69]
Breast cancer	miR-181c	Change the localization of TJs and actin filaments by inducing downregulation of PDPK1 gene in BMEC	in-vivo and in-vitro	[12]
Breast cancer	lncRNA GS1-600G8.5	Reduce the expression of TJs at BMEC	in-vitro	[115]
Breast cancer	CEMIP	Increase the vascular co-option	in-vitro	[117]
		Induce proinflammatory phenotype in BMEC	in-vivo	
Lung cancer	miR-550a-3-5p	Reduce expression of Yes-associated protein 1 in BMEC	in-vitro	[16]
Lung cancer	LINC01356	Reduce expression of TJs at BMEC	in-vitro	[122]
Lung cancer	Lnc-MMP2-2	Upregulate EPB41L5 that induces endothelial-to-Mesenchymal Transition and causes a reciprocal repression of BMECs TJs	in-vitro and in-vivo	[123]

**Table 3:** Clinical trials on the application of exosomes as therapeutics in CNS diseases.

Condition	Trial Description	Interventions	Phase	Study Status	Enrollment	NCT Number
Cerebrovascular Disorders	Using MSC exosomes to improve functional recovery in poststroke patients.	MSC exosomes	Phase 1, Phase 2	Unknown	N = 5	NCT03384433
Refractory Focal Epilepsy	Assess the safety, efficacy, and tolerability of nasal drops containing induced pluripotent stem cell (IPSC) exosomes in focal refractory epilepsy therapy.	IPSC exosomes	Early Phase 1	Recruiting	N = 34	NCT05886205
Post-stroke Dementia	Investigating the significance of acupuncture-induced exosomes in post-stroke dementia therapy.	Device: Acupuncture	Not applicable	Recruiting	N = 30	NCT05326724
Alzheimer Disease	Assessing the efficacy and safety of allogenic adipose mesenchymal stem cell exosomes in AD patients with dementia.	low, mild, and high dosage MSC Exosomes administrated for nasal drip	Phase 1, Phase 2	Unknown Status	N = 9	NCT04388982
Malignant Glioma of Brain	Activating the patient’s immune system using apoptosis released tumor exosomes loaded with tumor antigens.	Drug: IGF-1R/AS ODN	Phase 1	Completed	N = 13	NCT01550523
		Device: biodiffusion chamber				

## Data Availability

Not applicable.
